# Sirtuin1: A Promising Serum Protein Marker for Early Detection of Alzheimer’s Disease

**DOI:** 10.1371/journal.pone.0061560

**Published:** 2013-04-16

**Authors:** Rahul Kumar, Prasun Chaterjee, Prakash K. Sharma, Abhay K. Singh, Abhishek Gupta, Kamaldeep Gill, Manjari Tripathi, Aparajit B. Dey, Sharmistha Dey

**Affiliations:** 1 Department of Biophysics, All India Institute of Medical Sciences, New Delhi, India; 2 Department of Geriatric Medicine, All India Institute of Medical Sciences, New Delhi, India; 3 Department of Neurology, All India Institute of Medical Sciences, New Delhi, India; Rush University, United States of America

## Abstract

Sirtuin (SIRT) pathway has a crucial role in Alzheimer’s disease (AD). The present study evaluated the alterations in serum sirtuin1 (SIRT1) concentration in healthy individuals (young and old) and patients with AD and mild cognitive impairment (MCI). Blood samples were collected from 40 AD and 9 MCI patients as cases and 22 young healthy adults and 22 healthy elderly individuals as controls. Serum SIRT1 was estimated by Surface Plasmon Resonance (SPR), Western Blot and Enzyme Linked Immunosorbent Assay (ELISA). A significant (p<0.0001) decline in SIRT1 concentration was observed in patients with AD (2.27±0.46 ng/µl) and MCI (3.64±0.15 ng/µl) compared to healthy elderly individuals (4.82±0.4 ng/µl). The serum SIRT1 concentration in healthy elderly was also significantly lower (p<0.0001) compared to young healthy controls (8.16±0.87 ng/µl). This study, first of its kind, has demonstrated, decline in serum concentration of SIRT1 in healthy individuals as they age. In patients with AD and MCI the decline was even more pronounced, which provides an opportunity to develop this protein as a predictive marker of AD in early stages with suitable cut off values.

## Introduction

Alzheimer’s disease (AD), a neurodegenerative disease and leading cause of dementia, has emerged as a major public health challenge for ageing populations all over the world. The characteristic pathological changes of irreversible neuronal loss; and deposition of plaques laden with amyloid-β peptide; and neurofibrillary tangles made up of abnormally hyperphosphorylated tau protein in critical areas of brain have been well established [1], [2]. The poor understanding of the pathogenesis of AD and consequent lack of definitive therapy provides opportunity for development of newer diagnostic and treatment strategies. In this scenario recent studies have indicated a possible mechanism involving the sirtuin proteins which may have diagnostic and therapeutic potential in AD.

Sirtuins are NAD-dependent deacetylases, which have wide spectrum of metabolic and stress-tolerance properties. Among them SIRT1 is well characterized and is considered to be responsible for delaying the process of ageing in animal models [3]. SIRT1 has also been credited to have neuro-protective action against stress in cell cultures [4]. Calorie restriction, which protects experimental animals from neurodegenerative diseases, including AD [5], has shown to be mediated by SIRT1 [6]. Its therapeutic potential, against AD in transgenic mouse model over-expressing SIRT1, has been reported [7]. The exact role of SIRT1 in prevention of AD in animal models is not clear. It has been reported that SIRT1 increases the expression of ADAM10 gene encoding α secretase which protects against accumulation of pathogenic Aβ peptide [8].

The significant decrease in SIRT1 concentration in parietal cortex in autopsy specimens of AD patients was reported earlier and a strong correlation was established between tissue SIRT1 concentration, duration of symptoms and tau accumulation, but the exact relationship and its role in the sequence of events leading to development of AD remains unclear [9].

Though SIRT1 has been found to be promising in animal models of AD and autopsy specimens, its value in clinical setting remains an unexplored area. In the present study, estimation of serum SIRT1 concentration in patients with AD and MCI; and young and elderly controls was carried out to explore if there is any clinical relevance of what has been observed in animal models and autopsy specimen. There have been attempts to develop diagnostic markers for AD, which are reliable and not dependent on cerebrospinal fluid (CSF) or brain tissue. However, there are virtually no biomarkers for AD till date which can be reliably estimated in blood samples. In this setting the value of SIRT1 as biomarker of AD would be examined.

## Materials and Methods

### Cases

Forty patients suffering from AD and nine patients with MCI were recruited from the Geriatric Medicine OPD and Neurology OPD of All India Institute of Medical Sciences hospital, New Delhi, India. The Ethics Committee of AIIMS approved the study protocol (IESC/T-270/01.07.2011) and informed consent was obtained. The study was performed compliant to the rules and regulations of the Ethics Committee, all subjects gave written informed consent. The diagnosis of AD involved a two-step diagnostic process: screening for cognitive impairment with Folstein’s Mini Mental State Examination scale (MMSE; scores ≤24) and confirmation by a detailed neurological examination, assessment of activities of daily living and neuro-psychological testing using Clinical Dementia Rating Scale and the Blessed Dementia rating Scale. AD was diagnosed as per NINCDS-ADRDA criteria. MCI was diagnosed in patients with memory complaints and MMSE scores above 24 with normal Clinical Dementia Rating Scale and the Blessed Dementia Rating Scale but abnormalities in memory domain of Wechsler Adult Intelligence Scale (WAIS), Rey-Osterrieth Complex Figure for visuospatial competence and auditory visual learning tests.

### Controls

Young adults amongst post-graduate students of the department in normal health and elderly individuals (above 65 years) in good health (no obvious disease or disability in clinical examination and normal MMSE scores) were invited to participate in the study. 22 from each group were recruited as controls.

### Estimation of Serum SIRT1 Concentration

Two ml of venous blood was collected from each individual in vaccutainers under strict aseptic conditions. The serum was separated after centrifugation of clotted blood at 3000 rpm for 20 minutes.

#### By Surface Plasmon Resonance (SPR)

All SPR measurements were performed at 25°C using the BIAcore-2000 [Pharmacia Biosensor AB, Uppsala, Sweden] which is a biosensor-based system for real time specific interaction analysis. IgG primary mouse monoclonal antibody against SIRT1 [Santa Cruz Biotechnology, CA] of human origin was immobilized on the CM5 sensor chip using the amine coupling kit [Pharmacia Biosensor AB,Sweden]. The standard curve was prepared by passing 6 different concentrations of purified recombinant SIRT1 protein (0.62, 3.12, 6.25, 18.75, 31.25, and 62.25 ng/µl) over the immobilized antibody and corresponding resonance units (RU) obtained. SIRT1 protein was expressed and purified in bacterial system as described earlier [10]. Similarly, serum was passed over the immobilized SIRT1 antibody. The RU for each sample was recorded and the concentration of SIRT1 in patient serum was derived from the standard curve.

#### By western blot

To confirm the presence of SIRT1, serum samples from AD, MCI patients and controls were prepared by removing major interfering proteins by ‘plasma 7 multiple affinity removal spin cartridge according to the manufacturer’s protocol [Agilent Technologies, Santa Clara, CA]. total protein concentration was determined using Bicinchoninic acid assay (BCA). Standard protocol for Western Blot experiment was followed by using primary rabbit antihuman SIRT1 monoclonal IgG (1∶1000) and secondary HRP (Horse Radish Peroxidase) conjugated goat anti mouse IgG [GenScript, Piscataway,NJ] and visualized by Enhanced Chemiluminescent System [Pierce ECLWestern Blotting Substrate, Thermo Scientific, Rockford, IL]. The densities of the bands obtained were determined using the Quantity-one1-D-analysis software [Bio-Rad Laboratories, Hialeah FL].

#### By ELISA

Microtiter plates were coated with equal amount of serum sample (50 µg) in each well and the standard protocol of ELISA was followed by using primary mouse anti-human SIRT1 monoclonal IgG and secondary Alkaline Phosphatase conjugated goat anti mouse IgG [Chemicon]. The formation of Nitrophenolate 158 was measured at 405 nm using ELISA reader [Quanta Biotech, UK]. Six different concentrations of purified SIRT1 (1, 3, 5, 7, and 9 ng/µL) were used to plot a standard curve.

### Statistical Analysis

Statistical analysis was carried out using the Graphpad Instat3 software and p<0.05 was considered statistically significant. For the comparison of the findings, paired and unpaired t-test was performed. ROC analysis was done by IBM SPSS **(**Statistical Product and Service Solutions) Statistics Software to determine the specificity and sensitivity of SIRT1 for MCI and AD patients.

## Results

### Demographic Data

Demographic details of cases and controls are provided in Table 1. Serum SIRT1 concentrations were highest in young volunteers which declined significantly in healthy elderly controls and the values were significantly lower in MCI and AD. The trend was similar in both SPR assay and ELISA. The serum sirtuin values by SPR in cases and controls are presented in Table 2.

**Table 1:** Demographic data of patients and controls.

	AD	MCI	Geriatric control	Young Control
N	40	9	22	22
Males	26	8	13	11
Females	14	1	9	11
Mean age(years)	74.10±6.80	69.67±4.71	72.5±5.62	26.68±3.77
Mean MMSE	17.75±4.26	25.67±1.22	29.05±0.78	–
Mean Duration(years)	2.3±1.43	1.61±0.82	–	–
Mean Education(Years)	11.58±7.2	13.11±2.47	11.23±5.23	–
Rural	3	1	2	–

**Table 2:** Level of serum SIRT1 (ng/µl) in respect to demographic parameters by SPR.

Category	Subcategory	AD	MCI	Geriatric Control	Young Control
Age (Years)					
	65–75	2.21±0.48	3.68±0.11	4.86±0.46	–
	>75	2.34±0.44	3.35	4.71±0.29	–
Gender					
	M	2.3±0.43	3.65±0.16	4.78±0.32	8.4±0.51
	F	2.22±0.51	3.58	4.87±0.54	7.92±1.09
Duration (Years)					
	≤2	2.19±0.49	3.69±0.11	–	–
	>2	2.38±0.40	3.46±0.16	–	–
Education (Years)					
	0–10	2.29±0.33	3.63±0.07	4.82±0.45	–
	15-Oct	2.23±0.59	3.62±0.17	4.77±0.34	–
	>15	2.29±0.48	3.81	4.87±0.53	

### Quantitative Analysis of Serum SIRT1

#### By SPR

**Figure 1:**
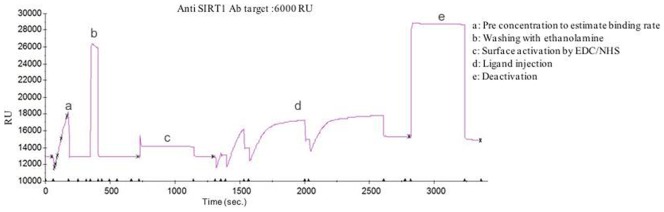
Sensogram showing the immobilization of SIRT1 antibody on CM5 sensor chip.

**Figure 2:**
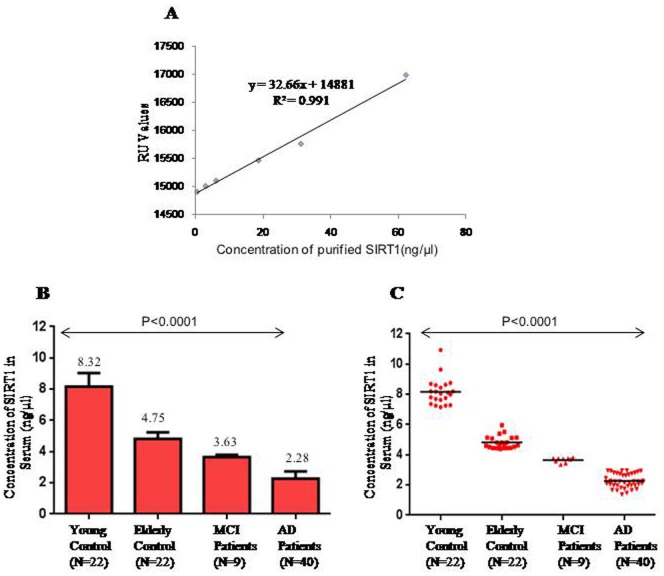
Estimation of serum SIRT1 using SPR technology. (A) Standard curve plotted between known concentration of SIRT1 and Resonance unit(RU). (B) Bar Diagram showing the difference in serum SIRT1 concentration between different groups (C) Scatter Diagram showing the difference in serum SIRT1 concentration between different groups.

The SPR signal for immobilization of human SIRT1 antibody was 7212.5 RU (Figure 1). The standard curve was plotted (Figure 2A) between RU obtained (14918.4, 15021.1, 15115.5, 15473.6, 15768.3, and 16982.0) and six different concentrations of pure SIRT1. The binding of the ligands i.e. SIRT1 was in the linear range. The RU increased linearly as the concentration of SIRT1 increased which provided evidence of sensitivity of the protein. The concentration of SIRT1 in serum was determined from the standard curve using RU obtained from binding of serum over the SIRT1 antibody. One RU corresponds to immobilized protein concentration of 1 pg/mm^2^. The concentration of SIRT1 in serum of young controls (8.16±0.87 ng/µl, 95% CI:7.77–8.55 ng/µl) was significantly higher (p<0.0001) than that of elderly controls (4.82±0.4 ng/µl, 95%CI: 4.63–5.0 ng/µl) (Figure 2B). In MCI patients a significant decline (p<0.0001) in serum SIRT1 concentration (3.64±0.15 ng/µl, 95% CI: 3.52–3.76 ng/µl ) (Figure 2C) was observed as compared to elderly controls. In AD patients, the decline in concentration of SIRT1 (2.27±0.46 ng/µl, 95% CI: 2.12–2.42 ng/µl ) was almost two fold compared that in elderly controls (<0.0001), (Figure 2D). Serum SIRT1 level was found to be significantly higher (p<0.0001) in MCI patients as compared to AD patients. A significant change (p<0.0001) in serum SIRT1 was observed between different groups i.e. elderly control, MCI patients, AD patients of age between 65–75 and above 75 yrs.

In all groups the SIRT1 concentrations were in a narrow range. The values in young controls were between 7.16 and 10.93 ng/µl, in elderly controls 4.41 and 5.96 ng/µl, in MCI patients the concentration were between 3.35 and 3.81 ng/µl and finally in AD patients the concentrations of SIRT1 were in the range of 1.37 and 2.99 ng/µl.

In case of AD, the sensitivity and specificity of SIRT1 were 95% and 100% respectively at the cut-off value of ≤2.94 ng/µl, while compared to healthy elderly control. In MCI patients at the cut of ≤3.78 ng/µl, the sensitivity and specificity of SIRT1 were 89% and 100% respectively compared to elderly control.

No significance difference was observed in serum SIRT1 concentration with respect to age, sex, years of education and duration of disease among AD patients (Table 2). A significant correlation (p<0.0001) existed between serum SIRT1 concentration and MMSE scores in elderly control (MMSE>28), MCI patients (MMSE 24–27) and AD patients (MMSE<24).

#### By western blot

**Figure 3:**
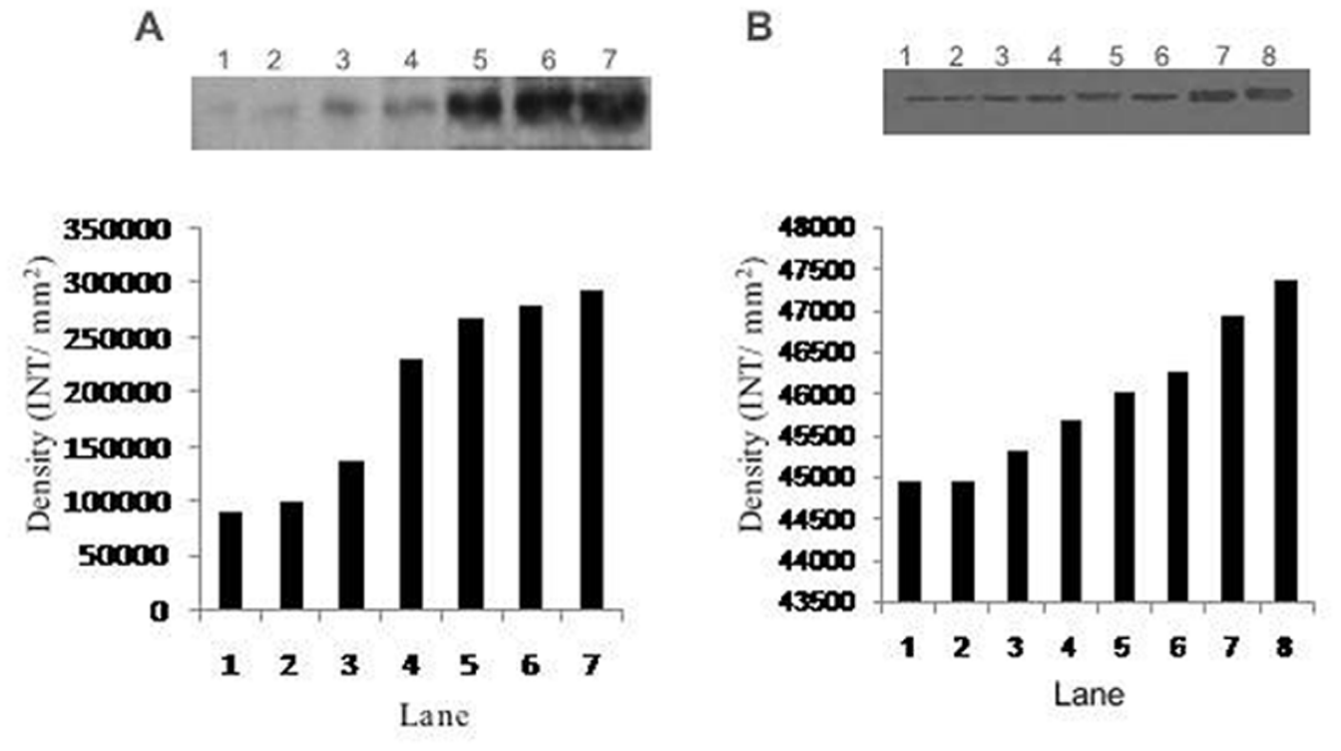
Western blot and density analysis (A) to confirm the presence of SIRT1 in serum of AD patients(lane1,2), MCI patients(lane3,4), Elderly control(5,6) and young control(7,8). (B) 1,3,9,12,15,18 and 21 µg of pure SIRT1 loaded in lane 1–7 respectively.

Western Blot estimation of SIRT1 in serum showed the low density band in MCI and AD patients as compared to elderly and young healthy controls (Figure 3A). The linearity of the sensitivity and specificity of the SIRT1 antibody are illustrated in Figure 3B.

#### By ELISA

**Figure 4:**
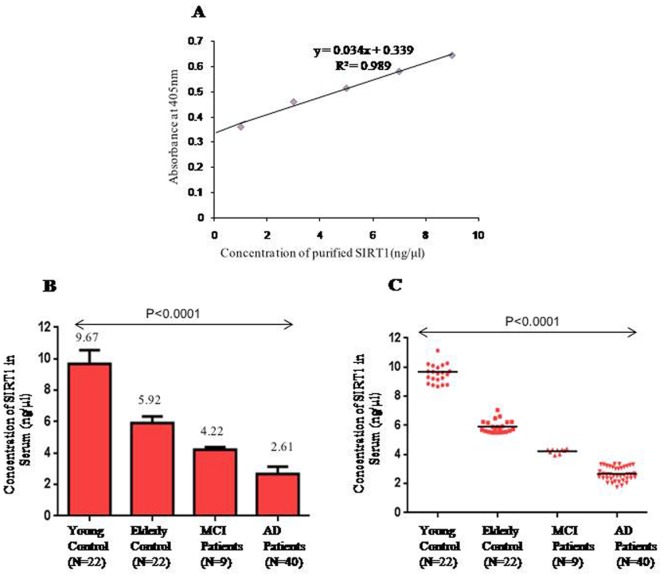
Estimation of serum SIRT1 using ELISA technology. (A) Standard curve plotted between known concentration of SIRT1 and absorbance obtained at 450 nm. (B) Bar Diagram showing the difference in serum SIRT1 concentration between different groups (C) Scatter Diagram showing the difference in serum SIRT1 concentration between different groups.

The concentration of SIRT1 in serum was determined using standard curve (Figure 4A). The concentrations were 9.67±0.87 ng/µl (95%CI:9.28–10.26 ng/µl) for young controls, 5.92±0.41 ng/µl (95%CI: 5.73–6.10 ng/µl) for elderly controls, 4.22±0.15 ng/µl (95%CI: 4.10–4.34 ng/µl) for MCI patients and 2.67±0.14 ng/µl (95%CI: 2.52–2.82 ng/µl) for AD patients (Figure 4B,C,D). The results obtained followed the same pattern as shown by SPR data.

## Discussion

Dementia and specifically Alzheimer’s disease is a major health issue for the ageing populations in terms of number, difficulty in detection in early stage, lack of definitive therapy, need for prolonged nursing care and economic cost. It is a global health issue and a rapidly ageing country like India is also experiencing the challenges of dementia care. In India 3.7 million people are estimated to be suffering from dementia and among them 2.2 million are women and 1.5 million are men [11]. In the past 100 years since Alzheimer’s disease was described in the early part of twentieth century, scientific knowledge regarding the condition has increased albeit incompletely. Till date the exact sequence of events of degenerative changes in the brain leading to AD has not been clearly described. The disease is generally detected after critical affection of cognitive capacity leaving little scope for reversal. Thus the therapeutic strategies for AD remain symptomatic and unsatisfactory. The descriptions of Mild Cognitive Impairment in 1990 s [12] have provided some window of opportunity for intervention if detected early for progression of this condition to AD [13].

Ageing has been established as the strongest risk factor for developing MCI and AD in epidemiological studies all over the world. Progressive increase in the prevalence of AD with increasing age (doubling every 5 to 6 years after the age of 60 up to 9^th^ decade) [14] supports the notion that AD may be a state of accelerated ageing in term degenerative changes in the brain. This leads one to consider if anti-ageing interventions can be of preventive value in dealing with AD in an ageing populations. Of all the anti ageing interventions described in animal models, calorie restriction has been the most effective in terms of longevity and prevention of age associated illnesses [15].

The role of sirtuins in prevention of brain degeneration especially in AD has been reported. SIRT1 increases the expression of ADAM10 gene encoding α secretase. In case of AD the down regulation of SIRT1 reduces the expression of α-secretase and as a result the accumulation of pathogenic Aβ peptide formed by β and γ secretase [16]. The serum Aβ1–40 levels is higher in the AD group than both controls and MCI. It can correlate that as SIRT1 down regulate in AD which control the expression of Aβ peptide through ADAM10 hereby upregulated the level of Aβ peptide [17]. Table 3 compares the serum SIRT1 level with Aβ peptide of AD. The tau protein is an established CSF biomarkers for AD. Till date no data is available on tau in serum or plasma of AD or MCI [18].

**Table 3:** Comparison of Serum SIRT level with that of Aβ previously quantified in serum.

	Controls	MCI Patients	AD Patients	Reference
SIRT1	4.82±0.4 ng/µl	3.64±0.15 ng/µl	2.27±0.46	Present Study
Aβ1–40	158±7.65 pg/ml	158±17.55 pg/ml	181±13.78 pg/ml	[17]
Aβ1–42	10±1.84 pg/ml	23±5.93 pg/ml	13.89±2.00 pg/ml	[17]

It has also been reported that production of β -amyloid plaques in mouse models of AD can be reduced by over expressing the NAD-dependent deacetylase SIRT1 in brain [19]. Resveratrol, the SIRT1 activator, has proved to be beneficial *in vitro* and *in vivo* rat model of AD, reducing amyloid-*β* protein accumulation [20].

Aβ plaque deposition and neurodegeneration in AD is maximum in the regions that metabolize glucose by aerobic glycolysis [21]. Incidentally aerobic glycolysis also involves a gradual depletion of NAD+ reserves within the cells through increased NADH production and decreased NAD^+^ regeneration through oxidation. SIRT1 deacetylase activity is inhibited by decrease in NAD^+^ [22], which results in a shift of APP processing towards the amyloidogenic pathway [23].

Demonstration of low SIRT1 concentration in brain tissue of AD patients in autopsy specimens, which correlated with duration of symptoms and tau accumulation, provides clinical relevance of the above observations in animal experiments [8]. The present study adds another perspective to these results by demonstrating declining SIRT1 concentration in blood in living patients. SIRT1 concentrations declines with age and the decline was most marked in cases of AD and somehow less marked (though significant) in patients with MCI. So this difference of SIRT1 level can give an indication of early detection of AD. The results tend to suggest that MCI and AD behave as situations of accelerated ageing if SIRT1 concentration is considered as the indicator.

The present study involved SPR technology which was further confirmed by traditional method like ELISA and Western Blot. SPR is a label free real time assay and its advantage over other immunological methods such as ELISA, lies in its reusability. The high sensitivity and specificity of the technology in detecting SIRT1 protein was also established.

One of the most promising approaches to identify potential protein markers for a disease is by analyzing human body fluid (e.g. blood, urine, saliva etc.) proteome. Human serum proteins originate from a variety of tissues and enter the circulation as a result of secretion and leakage [24]. The concentration of these proteins reflect human physiological or pathological state as suggested by several earlier reports [25], [26]. The present study for the first time detected and evaluated SIRT1 protein in serum of AD and MCI cases in comparison to young and elderly controls which well with MMSE. In addition the values of serum SIRT1 concentration in different groups (cases and controls) were in a narrow range and did not change vary with gender and duration of disease.

Can SIRT1 evolve as a biomarker and can sirtuin be of any therapeutic value in MCI/AD? The steadily declining concentrations with age, values consistently within a narrow range and significant difference between MCI and AD, do indicate a possible clinical utility for SIRT1. It may be possible that the subsets of patients with MCI who are likely to transform to AD can be identified early with a degree of confidence. Sirtuin activators may also have a preventive role in these patients and early stage of AD as well. A longitudinal study with larger sample of MCI cases and non-AD dementias controls may required for further progress in this hypothesis, which would also detect the decline in SIRT1 concentration in AD cases with time.
